# Longitudinal Associations among Impulsivity, Friend Substance Use, and Adolescent Substance Use

**DOI:** 10.4172/2155-6105.1000220

**Published:** 2015-05-08

**Authors:** Julee P. Farley, Jungmeen Kim-Spoon

**Affiliations:** 1 Research Coordinator, Department of Psychology, Virginia Tech, VA, USA; 2 Associate Professor, Department of Psychology, Virginia Tech, VA, USA

**Keywords:** Longitudinal study, Peer influence, Regulation, Adolescent development, Risk-taking behavior

## Abstract

Adolescent substance use is an increasing problem in the United States, and some researchers posit a bidirectional relation between adolescent substance use and the personality trait of impulsivity (e.g., Quinn, Stappenbeck, & Fromme, 2011). Friend substance use has been shown to be a powerful predictor of adolescent substance use, with prior research suggesting a bidirectional relation between adolescent substance use and friend substance use (e.g., Simons-Morton & Chen, 2006). Extant literature has not tested the bidirectional relation between adolescent substance use and impulsivity with longitudinal data nor has it examined this relation while considering the bidirectional relation with the social context factor of friend substance use. Using three waves of longitudinal data, we tested if there was a bidirectional relation between adolescent substance use and impulsivity while also examining the influences of friend substance use. Participants were 131 adolescents (male = 55%, mean age = 13 years at Wave 1). We tested nested models and examined whether adding equality constraints degraded the model fit using a Wald test. Results of structural equation modeling indicated that, after controlling for baseline levels of substance use, impulsivity predicted adolescent and friend substance use over time, whereas adolescent and friend substance use did not predict impulsivity. Adolescents with substance using friends were likely to increase their own substance use. The findings imply that aiming at both improving adolescents’ ability to regulate impulsivity and deterring associations with friends who are using substances is essential for prevention and intervention efforts against substance use development in adolescents.

## Introduction

Given that among high school students in the United States, almost 45% have tried cigarettes, 70.8% have tried alcohol, and almost 40% have tried marijuana, drug use is becoming an increasing concern among adolescent populations [1]. In addition, drug abuse in the United States is estimated to cost approximately $510.8 billion a year [2]. Consequently, determining both the antecedents and consequences of adolescent substance use is crucial for both adolescent health and minimizing societal cost of drug use.

A debate exists in extant literature about the relation between substance use and the personality trait of impulsivity. Some researchers posit that impulsivity is a somewhat stable personality trait regardless of drug use [3], whereas other researchers contend that the relation between drug use and impulsivity is bidirectional [4]. Still other researchers joining this debate insist that we do not yet know if impulsivity is a unique predictor of substance use or if it is a joint predictor with other variables [5]. Friend substance use is another powerful predictor of adolescent substance use, and the bidirectional association between adolescent substance use and friend substance use has been shown in prior research [6–8]. Though existing literature has argued for the significant role of impulsivity in the development of adolescent substance use, to the authors’ best knowledge, it has not tested the bidirectional association between adolescent substance use and impulsivity with longitudinal data, nor has it concurrently examined this association while considering the bidirectional association with the social context factor of friend substance use.

In the current study, we utilize three waves of longitudinal data to test the bidirectional association between impulsivity and adolescent substance use. In addition, we compare the longitudinal bidirectional association between impulsivity and adolescent substance use to the longitudinal bidirectional association between friend and adolescent substance use. The main goal was to evaluate the relative developmental contributions of both a prominent individual, personality characteristic (i.e., impulsivity) and a prominent social context factor (i.e., friend substance use) that are associated with the development of adolescent substance use.

While there are many definitions of and ways to measure impulsivity [9,10], the present study examines impulsivity as a trait-like personality feature rather than using a more state-dependent measure [11]. Eysenck (1993) and more recent theorists [12,13] suggest that this trait-like impulsivity may be associated with failure to consider the consequences of actions which may then lead to engaging in risky behaviors like substance use. In addition, extant literature in college students and young adults suggest that there is a bi-directional relation between increases in heavy drinking and increases in impulsivity [14], but this relation may be temporally restricted to shorter spans of time [15]. However, these associations have not been examined in adolescents while simultaneously considering an important social context factor such as friend substance use. Given that adolescents’ initiation and experimentation with drugs and alcohol can readily result in long-term trajectories of health problems and addiction in adulthood [16], it is important to examine how adolescent personality, social context, and substance use interact in order to create effective prevention and intervention efforts for adolescent substance use.

Extant literature links impulsivity to substance use in adolescence and young adulthood. For example, in young adults high levels of impulsivity are related to a higher probability of engaging in habitual smoking behaviors [17]. Similarly, adolescent smokers are found to be more impulsive than nonsmokers in both questionnaire and behavioral measures [18]. Across the lifespan, impulsive people are more likely to enjoy consuming alcohol and engage in heavy levels of alcohol consumption [19]. Adolescent marijuana users have also been found to be more impulsive than are non-using adolescents [20]. Although evidence of the positive association between impulsivity and adolescent substance use exists, to date, we do not have a clear understanding as to whether impulsivity contributes to developmental changes in adolescent substance use and vice versa, thus the present study seeks to examine dynamic cross-lagged associations between impulsivity and substance use among adolescents.

When examining the findings of prior studies, it is apparent that high levels of impulsivity are related to high levels of substance use across many types of substances. However, research examining effects of substance use on impulsivity are rarer and often use retrospective data. For example, an earlier age of onset of marijuana use in adolescents is associated with poorer performance on executive function tasks, possibly because using marijuana during early brain development may alter brain development [21]. While there is evidence for a general trend to “mature out” of problematic alcohol use [22], there is also evidence for the existence of a smaller group of people who continue to have high impulsivity and alcohol use [23]. The present study seeks to extend the prior research by examining a longitudinal and bidirectional relation between adolescent substance use and impulsivity to examine developmental changes in the association between these variables.

Associations between the adolescent personality trait of impulsivity and friend substance use have not been examined extensively in existing literature. However, available evidence suggests that adolescent impulsivity levels may be related to levels of substance use in friends. For example, adolescents who are more impulsive are more likely to have friends who engage in alcohol use compared to less impulsive adolescents [19]. In addition, friends’ support of substance use is negatively associated with better impulse control shown as higher effortful control [24]. In their review, [25] concluded that in general good self-control is associated with fewer deviant peer associations. Thus, it appears that those adolescents who are more impulsive tend to have friends who engage in more substance use. The present study extends prior research findings by examining the effects of impulsivity in tandem with adolescent substance use on friend substance use as well as longitudinal contributions of friend substance use to the developmental changes in impulsivity and substance use.

Previous research suggests a bidirectional association between adolescent substance use and selection of friend groups who also use substances [6–8]. While adolescents who use substances are more likely to select friends who also use substances, having friends who use substances also contributes to adolescent substance use. There is also evidence that this similarity between adolescent substance use and friend substance use lasts into young adulthood [26,7]. The relation between adolescent substance use and friend substance use may exist because adolescents are rewarded socially for engaging in similar levels of substance use as their peers do [27]. Furthermore, research indicates that adolescents who smoke are more susceptible to the influence of peers on risk-taking behavior than are adolescents who do not smoke [28], suggesting that more substance using adolescents are more susceptible to peer influence than are adolescents who do not use substances. This relationship does not occur because deviant adolescents are more accepted by their peers; adolescents who are consistently delinquent are not more accepted by their peers [29]. Rather, it seems that peer contagion is the mechanism through which much of peer influence occurs [30]. We further extend the previous research by investigating the contribution of friend substance use not only to adolescent substance use, but also to adolescent impulsivity.

### The present study

To the authors’ knowledge, this study is the first to examine this bidirectional relation between adolescent substance use and impulsivity in the context of friend substance use using multiple waves of longitudinal data. Adolescent substance use was measured at Waves 1, 2, and 3, and impulsivity and friend substance use were measured at Waves 2 and 3. We hypothesized bidirectional associations between adolescent substance use and the two predictors of impulsivity and friend substance use as follows: Higher adolescent substance use at Wave 1 would be related to higher adolescent substance use, friend substance use, and adolescent impulsivity at Wave 2. Higher adolescent substance use at Wave 2 would be related to increases in adolescent substance use, friend substance use, and adolescent impulsivity at Wave 3. Higher friend substance use at Wave 2 would be related to increases in adolescent substance use, friend substance use, and adolescent impulsivity at Wave 3. Finally, higher adolescent impulsivity at Wave 2 would be related to increases in adolescent substance use, friend substance use, and adolescent impulsivity at Wave 3.

## Method

### Participants

Participants were part of a longitudinal research study on risk and protective processes related to adolescent health risk behaviors. At Wave 1, participants included 357 adolescents between the ages of 10 to 17 years (*M* = 13.03, *SD* = 1.91). A total of 220 adolescents (121 males) participated approximately two years later (Wave 2) and were between the ages of 11 to 18 years (*M* = 15.12, *SD* = 1.56). In wave 3 of the study, which was conducted about two years after Wave 2, 167 adolescents (90 males) between the ages of 13 to 21 years (*M* = 17.13, *SD* = 1.65) participated. The final sample size for the current study was 131 adolescents who had participated in all three waves of the study and consisted of 50% male and 93% White followed by 7% reporting themselves as African-American, Hispanic, or other races. As for parents’ marital status, 78% of parents were married while 22% were separated, divorced, widowed, or never married. Unlike Waves 1 and 2 in which parents reported family demographic information, only adolescent reports were collected at Wave 3, and no family income information was available. At both Waves 1 and 2, family income ranged from no source of income to earning more than $200,000 a year and mean family income was between $50,000 and $74,999 a year for the final sample.

## Procedures

For the first wave of the study, participants were recruited from areas in a southeastern state via letters using address lists purchased from contact companies, email announcements, flyers, notices placed on the internet, or snowball sampling (word-of-mouth). For the second wave of this study participants were contacted about two years after their first participation. For both Waves 1 and 2, adolescents and their parents were interviewed privately and simultaneously, and received monetary compensation. Those who had already attended their first year of college were aged out of Wave 2 of the study and were not asked to complete the study a second time. There were 137 participants that did not return for Wave 2 for reasons including: child not invited back due to age or other issues (n = 24), too busy (n = 8), moved away (n = 12), unable to reach (n = 86), child not interested (n = 6), and child death (n = 1). The third wave of this study was conducted about two years after the second wave. All those who participated in Wave 1 of the study were asked to participate in Wave 3 of the study regardless of their participation in Wave 2. A total of 167 adolescents completed Wave 3 of the study and received monetary compensation. Of the 190 adolescents who did not complete Wave 3, 55 adolescents were contacted but did not complete the survey while the remaining 135 adolescents were unable to be contacted or did not respond. Adolescents who participated in the third wave of the study completed an online survey and received monetary compensation. All procedures were approved by the institutional review board of the university.

We performed multivariate general linear modeling (GLM) analyses to determine if the final sample of 131 adolescents (who participated in all three waves) differed from the 226 adolescents who did not participate in all three waves of the study regarding on demographic variables as well as substance use variables at Wave 1. Results indicated that adolescents completed all three waves of the study had a higher average family income (*p* = .01) and were younger (*p* = .01), but the two groups had similar race (*p* = .06) and gender compositions (*p* = .89). However, the effect sizes of the attrition effects were small (η^2^ = .02 for income and η^2^ = .03 for age), and the sample had the family income level and the percentage of non-White persons that were representative of the Southwestern Virginia region that the data were collected (U.S. Census Bureau, 2011). Those adolescents who participated in all three waves of the study did not differ in levels of substance use (cigarette, alcohol, or marijuana) compared to those who did not complete all there waves of the study (*p* = .17 ~ .92).

### Measures

#### Adolescent substance use

At all three waves of the study, adolescents filled out a web-based computerized questionnaire regarding their use of cigarettes, alcohol, and marijuana. A composite of mean typical substance use of cigarettes, alcohol, and marijuana was utilized in the present study (e.g., “Which is most true for you about smoking cigarettes?”). Adolescent participants answered separately for typical frequency of use in each drug category (*1* = Never used to *6* = Usually use every day), and the mean was taken across the use of cigarettes, alcohol, and marijuana. In the current sample, reliability (Cronbach’s alpha) was .74 for Wave 1, .83 for Wave 2, and .81 for Wave 3.

#### Adolescent impulsivity

At both Waves 2 and 3 of the study, adolescent impulsivity was measured using adolescent self-report on the Eysenck Junior Impulsivity Scale [31]. This scale consists of 23 items asking if the question describes them (e.g., “Do you often do things on the spur of the moment?”). Adolescent response options were yes (*1*) or no (*0*), and the mean value of all responses was utilized. In the current sample, reliability (Cronbach’s alpha) was .83 for Wave 2 and .86 for Wave 3.

#### 2 Friend substance use

At both Waves 2 and 3 of the study, adolescents were asked to report on the number of friends they had who used cigarettes, alcohol, and marijuana. Adolescents were asked to report about those individuals with whom they considered themselves a friend and answered separately for number of friends who used each drug type (*1* = None of my friends to *5* = More than 3 of my friends). The mean was taken combining the number of friends who used each drug type. In the current sample, reliability (Cronbach’s alpha) was .88 for Wave 2 and .82 for Wave 3.

### Data analysis strategy

Structural Equation Modeling (SEM) analyses were conducted using Mplus statistical software package [32]. In order to conduct rigorous comparisons of bidirectional effects, we tested several hierarchically nested models and examined whether adding equality constraints significantly degraded the model fit using a Wald test [33]. If a certain equality constraint did not degrade the model fit, we kept it in the subsequent models. First, we tested a configural invariance model in which all the parameters were freed across the two groups. Second, we tested bidirectional effects between adolescent impulsivity and adolescent substance use. We constrained the path between adolescent impulsivity at Wave 2 and adolescent substance use at Wave 3 and the path between adolescent substance use at Wave 2 and adolescent impulsivity at Wave 3 to be equal. Third, to test bidirectional effects between adolescent impulsivity and friend substance use, we constrained the path between adolescent impulsivity at Wave 2 and friend substance use at Wave 3 and the path between friend substance use at Wave 2 and adolescent impulsivity at Wave 3 to be equal. Finally, to test bidirectional effects between friend substance use and adolescent substance use, we constrained the path between friend substance use at Wave 2 and adolescent substance use at Wave 3 and the path between adolescent substance use at Wave 2 and friend substance use at Wave 3 to be equal.

## Results

### Preliminary analysis

Table 1 presents bivariate correlations and descriptive statistics of study variables. We examined Wave 1 demographic statistics as possible covariates with study variables. Results of general linear modeling (GLM) analysis revealed that adolescent age was a significant predictor of adolescent substance use and friend substance use (*p* < .001) but not a significant predictor of adolescent impulsivity (*p* = .78). Other demographic variables, including adolescent gender (0 = male, 1 = female), adolescent race (0 = Caucasian, 1 = Ethnic/Racial minority), family income (1 = None, 15 = $200,000 +), and parent marital status (0 = married; 1 = never married, divorced, separated), were not significant predictors of adolescent impulsivity, friend substance use, and adolescent substance use (*p* = .21 – .89). Therefore, adolescent age was included as a covariate in all SEM analyses. All study variables were examined for normality. All variables, except adolescent substance use at Wave 1, were within an acceptable range (skewness less than 3 and kurtosis less than 10) [34] most likely because the incidence of substance use was low at Wave 1 in our sample due to younger ages. Log-transformed values of Wave 1 adolescent substance use were utilized in SEM analyses.

### Hypothesis testing

The first model examined the associations among adolescent substance use at Wave 1 and adolescent substance use and impulsivity and friend substance use at Wave 2 as well as the relation between adolescent substance use and impulsivity and friend substance use at Wave 2 to adolescent substance use and impulsivity and friend substance use at Wave 3. Model fit statistics were χ^2^ = 2.12, *df* = 5, *p* = .83, CFI = 1.00, and RMSEA = .00, indicating good model fit.

Reciprocal effects were compared via nested model testing. When the path between adolescent impulsivity at Wave 2 and adolescent substance use at Wave 3 and the path between adolescent substance use at Wave 2 and adolescent impulsivity at Wave 3 were constrained to be equal, model fit was significantly worse (Wald test χ^2^ = 5.33, *df* = 1, *p* = .03). Thus, nested model testing indicated a significant difference in the magnitude of cross-lagged effects between adolescent impulsivity and substance use showing that the relation between adolescent impulsivity at Wave 2 and adolescent substance use at Wave 3 was significant (b = .051, *SE =* 0.22, *p* = .02), whereas the relation between adolescent substance use at Wave 2 and adolescent impulsivity at Wave 3 was not significant (b = −0.00, *SE =* 0.03, *p* = .89). Similarly, when the path between adolescent impulsivity at Wave 2 and friend substance use at Wave 3 and the path between friend substance use at Wave 2 and adolescent impulsivity at Wave 3 were constrained to be equal, model fit was significantly worse (Wald test χ^2^ = 5.06, *df* = 1, *p* = .02). Specifically, the relation between adolescent impulsivity at Wave 2 and friend substance use at Wave 3 was significant (b = 0.93, *SE =* 0.42, *p* = .03), whereas the path between friend substance use at Wave 2 and adolescent impulsivity at Wave 3 was not significant (b = −0.02, *SE =* 0.02, *p* = .26). These findings suggest that the longitudinal effects of adolescent impulsivity on friend and adolescent substance use were significantly stronger than the longitudinal effects of friend and adolescent substance use on impulsivity over time.

**Figure 1:**
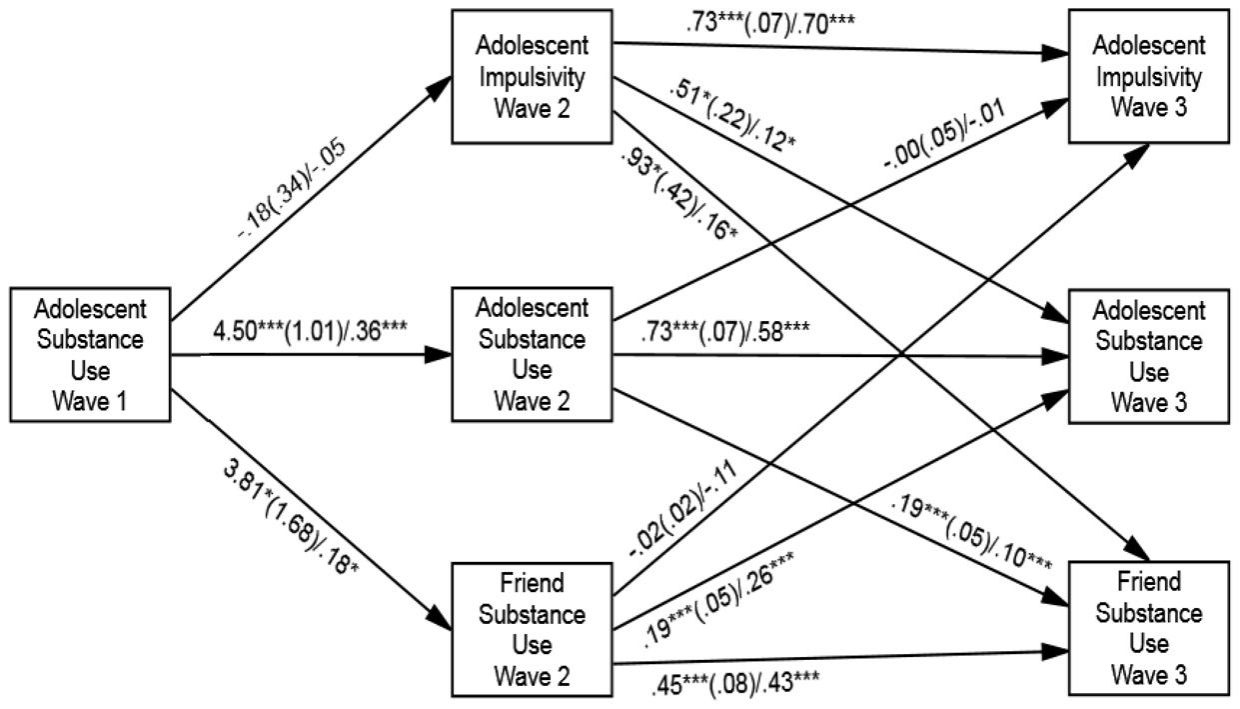
Results of the longitudinal associations among adolescent impulsivity, friend substance use, and adolescent substance use. Notes. Numbers on paths are unstandardized coefficient (SE)/standardized coefficient. For clarity of presentation, the age covariate as well as the following covariances among study variables are not shown: Friend substance use at Wave 2 ↔ Adolescent substance use at Wave 2 = .62***; Friend substance use at Wave 2 ↔ Adolescent impulsivity at Wave 2 = .40***; Adolescent impulsivity at Wave 2 ↔ Adolescent substance use at Wave 2 = .39***; Friend substance use at Wave 3 ↔ Adolescent substance use at Wave 3 = .45***; Friend substance use at Wave 3 ↔ Adolescent impulsivity at Wave 3 = .23**; Adolescent impulsivity at Wave 3 ↔ Adolescent substance use at Wave 3 = .22**; Age at Wave 1 → Adolescent substance use at Wave 1 = .29***; Age at Wave 1 → Adolescent substance use at Wave 2 = .23**; Age at Wave 1 → Adolescent substance use at Wave 3 = .02; Age at Wave 1 → Friend substance use at Wave 2 = .45***; Age at Wave 1 → Friend substance use at Wave 3 = .19** * p < .05; ** p < .01; ***p < .001.

When the path between friend substance use at Wave 2 and adolescent substance use at Wave 3 and the path between adolescent substance use at Wave 2 and friend substance use at Wave 3 were constrained to be equal, model fit was not significantly worse (Wald test χ^2^ = .01, *df* = 1, *p* = .92). These findings suggest that the reciprocal effects between friend substance use and adolescent substance use were comparable over time. Figure 1 presents the results of this final model with equality constraints between friend substance use at Wave 2 and adolescent substance use at Wave 3 and the path between adolescent substance use at Wave 2 and friend substance use at Wave 3. Model fit statistics of this final model were χ^2^ = 2.13, *df* = 6, *p* = .91, CFI = 1.00, and RMSEA = .00, indicating an excellent model fit. In the final model, adolescent substance use at Wave 1 was related significantly and positively to adolescent substance use and friend substance use at Wave 2 but not adolescent impulsivity at Wave 2. Adolescent substance use at Wave 2 was related significantly and positively to adolescent substance use and friend substance use at Wave 3 but not adolescent impulsivity at Wave 3. Adolescent impulsivity at Wave 2 was related significantly and positively to adolescent substance use and friend substance use at Wave 3 as well as adolescent impulsivity at Wave 3. Friend substance use at Wave 2 was related significantly and positively to adolescent substance use and friend substance use at Wave 3 but not adolescent impulsivity at Wave 3.

As a post-hoc test, we further examined whether there were developmental differences in the effects of adolescent substance use on friend selection between Wave 1 to Wave 2 and Wave 2 to Wave 3. Specifically, when the path between adolescent substance use at Wave 1 and friend substance use at Wave 2 and the path between adolescent substance use at Wave 2 and friend substance use at Wave 3 were constrained to be equal, model fit was significantly worse (Wald test χ^2^ = 5.98, *df* = 1, *p* = .01). Thus, the result of this post-hoc Wald test suggested that the association between adolescent substance use at Wave 1 and friend substance use at Wave 2 was significantly stronger (b = 3.81, *SE =* 1.68, *p* < .001) than the association between adolescent substance use at Wave 2 and friend substance use at Wave 3 (b = 0.19, *SE =* 0.05, *p* < .001).

## Discussion

This study examined longitudinal, dynamic associations among adolescent personality (impulsivity) and context (friend substance use) and substance use. In particular, this study represents the first longitudinal investigation of a bidirectional relation between adolescent impulsivity and substance use. We found significant longitudinal effects of impulsivity on adolescent and friend substance use but not vice versa, and found bidirectional associations between adolescent and friend substance use.

The results showed that impulsivity at Wave 2 predicted adolescent substance use at Wave 3 but that adolescent substance use at Wave 1 and Wave 2 did not predict impulsivity at Wave 2 and Wave 3, respectively. The findings demonstrate that impulsivity predicts increases in substance use, but that those adolescents who engage in substance use do not necessarily become more impulsive. Thus, the results of the present study indicate that the relation between adolescent impulsivity and substance use is not necessarily bidirectional. Our results suggest that impulsivity in adolescence and young adulthood is a somewhat stable personality trait [3] that is unchanged by drug use as some researchers suggest [4]. This result differs from prior research using a young adult sample that showed stronger effects of heavy drinking on changes in impulsivity compared to effects of impulsivity on changes in heavy drinking [14]. It may be that the significant bidirectional association is rather specific to heavy drinking. Further, the discrepancy in the findings may suggest possible dynamic changes in the association between impulsivity and substance use development between adolescence in which substance use patterns are still being established versus young adulthood in which substance use patterns are more established and stabilized. Taken together, these findings imply that substance use prevention and intervention programs designed for adolescents may need to focus on personality factors more so than the programs for adults would.

The present study also found that adolescents who are more impulsive are more likely to have a greater amount of friends who use substances. The results of the present study support the idea put forth by [19,25] that adolescents who are more impulsive select friends who are substance users. Also consistent with previous literature [6,26], we found that adolescents who use substances are more likely to have friends who use substances. Our rigorous statistical testing using nested model comparison further confirmed that the effect of adolescent impulsivity on friend substance use was significantly stronger than the effect of friend substance use on adolescent impulsivity over time. Thus, we saw a stronger effect of highly impulsive adolescents selecting substance-using friends rather than having substance-using friends prompting impulsivity in adolescents, and this result suggests that prevention and intervention efforts should focus on altering the choices of impulsive adolescents rather than controlling their social environment.

We found that the relation between adolescent substance use and friend substance use were bidirectional and comparable in magnitude. Specifically, adolescents with high levels of friend substance use at Wave 2 reported high levels of their own substance use at Wave 3. Similarly, adolescents with high levels of their own substance use at Wave 2 reported high levels of friend substance use at Wave 3. Thus, our results help understand why adolescents who use substances may be more susceptible to peer influences on risky behaviors [28]; adolescents who are high in impulsivity – and thus low on planning or considering the consequences of their actions – may be more susceptible to both positive and negative suggestions by peers. When considering the patterns of longitudinal associations among adolescent impulsivity and substance use and friend substance use, we see that adolescents who are at-risk for substance use due to impulsivity are also more likely to associate with substance using friends. Consistent with existing research using adolescent populations [8], this finding suggests “selection effects” demonstrating that adolescents who use substances seek out peers who are similar to them in behaviors.

In addition, we found that higher friend substance use at Wave 2 was related to higher adolescent substance use at Wave 3. This result suggests “socialization effects” indicating that adolescents with substance using peers are more likely to be afforded opportunities to use substances or see substance use as more normative. Thus, our findings provide evidence for both socialization and selection effects in adolescents similar to research by [8]. In contrast, [7] found evidence for socialization, but not selection, effects in their sample spanning from late adolescence into adulthood, suggesting that there may be some developmental trends in the strength of socialization and selection effects as adolescents age. Indeed, we found significant developmental differences in the longitudinal associations between earlier adolescent substance use and later friend substance use. Our finding indicated that adolescents’ niche-picking in substance use (i.e., selection effects) tends to be stronger among younger adolescents compared to older adolescents. This finding is consistent with previous research suggesting that as adolescents age they conform less to peer influences [35]. Thus, effective prevention and intervention efforts for adolescent substance use may need to be designed and targeted to specific age groups of adolescents in order to be most effective.

Our results highlight that adolescent impulsive personality is a mechanism through which this enhanced susceptibility to peer influence occurs. Thus, teaching adolescents regulatory skills to manage their impulsivity could be a possible route for intervention and prevention of adolescent drug use. In addition, targeting adolescent social groups and providing social reinforcement for non-drug using peers may provide a clear social incentive for adolescents not to use drugs. Adolescents seem to be rewarded by their peers for engaging in some level of drug use [27]. However, [12] suggests that learning societal norms may help engage the adolescent regulatory system, a view that appears to be supported by the “maturing out” hypothesis [22]. Therefore, future research should seek to untangle the influence of adolescent neurological maturation, the learning of social standards, and friend substance use on substance use in adolescent and young adult populations.

Finally, the present study extended the examination of the longitudinal relationships among impulsivity, friend substance use, and adolescent substance use to an understudied Appalachian population. Although the population utilized in the present study was not highly diverse, the sample composition is typical of the Appalachian area in which it was collected [36]. This rural area is understudied, and as the per capita income in the Appalachian region is only 68% of the national average and approximately 18% of individuals are below the poverty line [37], this region provides an important geographical area to study the relationships between impulsivity, friend substance use, and adolescent substance use. Nevertheless, generalizability of the findings to the families from diverse ethnic and socioeconomic groups awaits further study.

Some limitations of the present study should be noted. Primarily, we did not have impulsivity or friend substance use data for Wave 1 of the study. Therefore, we were unable to examine the full longitudinal and bidirectional influence of all study variables. Second, our final sample that completed all three waves of the study was more likely to be Caucasian, younger, and had higher family income than the adolescents who did not participate in all three waves of the study. This sample attrition, as well as the homogenous race composition of the sample, limits the generalizability of our findings to more ethnically diverse populations. Future research should examine this model in more racially and ethnically diverse samples. In addition, adolescents in the final sample that completed the study were also more likely to smoke cigarettes. Furthermore, we utilized gender as a covariate in our model, but we were not able to test gender moderation due to the small sample size; future studies replicating these findings with larger samples should consider examining possible gender differences in the patterns of the associations among impulsivity, friend substance use, and adolescent substance use. Finally, we have relatively low levels of substance use in our community sample. Therefore, future studies should examine longitudinal associations among adolescent substance use and impulsivity and friend substance use using substance abusing or addicted samples to evaluate generalizability of the current findings to adolescents with differing levels of substance use problems.

Despite the limitations, the current findings provide a unique look at the associations between adolescent impulsivity, substance use, and friend substance use. Using three waves of longitudinal data, we examined prospective, bidirectional associations among adolescent impulsivity and substance use and friend substance use. The current findings provided important insights about the antecedents and consequences of adolescent substance use as well as implications for preventing and treating adolescent substance use. The finding underscores that it may be beneficial to target both personality and context factors for the prevention and intervention of adolescent substance use. Specifically, aiming at both improving adolescents’ ability to regulate impulsivity and deterring associations with friends who are using substances may be essential for preventive intervention efforts against substance use development in adolescent populations.

## Figures and Tables

**Table 1:** Descriptive statistics and bivariate correlations of adolescent age, substance use, and impulsivity, and friend substance use.

	*M*	*SD*	2	3	4	5	6	7	8
1. Adolescent Age Wave 1	12.67	1.46	.30***	.30***	.48***	−.09***	.32***	.42***	−.07***
2. Adolescent Substance Use Wave 1	1.05	0.17		.42***	.31***	−.06***	.33***	.19***	−.05***
3. Adolescent Substance Use Wave 2	1.41	0.70			.68***	.30***	.80***	.50***	.12***
4. Friend Substance Use Wave 2	1.88	1.20				.28***	.70***	.63***	.08***
5. Adolescent Impulsivity Wave 2	0.38	0.22					.37***	.30***	.66***
6. Adolescent Substance Use Wave 3	1.61	0.88						.69***	.26***
7. Friend Substance Use Wave 3	2.53	1.27							.27***
8. Adolescent Impulsivity Wave 3	0.37	0.23							

* *p* < .05;

** *p* < .01;

*** *p* < .001.
